# 2D and 3D-QSAR study on 4-anilinoquinozaline derivatives as potent apoptosis inducer and efficacious anticancer agent

**DOI:** 10.1186/2191-2858-1-13

**Published:** 2011-10-04

**Authors:** Vivek Kumar Vyas, Manjunath Ghate, Hitesh Katariya

**Affiliations:** 1 Department of Pharmaceutical Chemistry, Institute of Pharmacy, Nirma University, S.G. Highway, Chharodi, Ahmedabad 382 481, Gujarat, India

**Keywords:** 4-anilinoquinozaline, apoptosis inducer, anticancer, QSAR, multiple linear regression (MLR), principle component analysis (PCA), partial least square (PLS), k-nearest neighbor molecular field analysis (kNN-MFA)

## Abstract

**Background:**

Apoptosis is known as programmed cell death that plays an important role in tumor biology.

**Methods:**

In this study, apoptosis-inducing activity is predicted by using a QSAR modeling approach for a series of 4-anilinoquinozaline derivatives. 2D-QSAR model for the prediction of apoptosis-inducing activity was obtained by applying multiple linear regression giving *r*^2 ^= 0.8225 and *q*^2 ^= 0.7626, principal component regression giving *r*^2 ^= 0.7539 and *q*^2 ^= 0.6669 and partial least squares giving *r*^2 ^= 0.8237 and *q*^2 ^= 0.6224.

**Results:**

QSAR study revealed that alignment-independent descriptors and distance-based topology index are the most important descriptors in predicting apoptosis-inducing activity. 3D-QSAR study was performed using k-nearest neighbor molecular field analysis (kNN-MFA) approach for both electrostatic and steric fields. Three different kNN-MFA 3D-QSAR methods (SW-FB, SA, and GA) were used for the development of models and tested successfully for internal (*q*^2 ^> 0.62) and external (predictive *r*^2 ^> 0.52) validation criteria. Thus, 3D-QSAR models showed that electrostatic effects dominantly determine the binding affinities.

**Conclusions:**

The QSAR models developed in this study would be useful for the development of new apoptosis inducer as anticancer agents.

## 1. Introduction

Cancer is a disease of cell characterized by progressive, persistent, abnormal, purposeless, and uncontrolled proliferation of tissues. Currently, cancer is most dominating cause of death in world [1,2]. Apoptosis is a cellular process, that organisms use for digestion of excessive cells to control cell numbers. Caspases is a family of cysteine proteases, which are produced as zymogenes, plays a vital role in the beginning and ending of apoptosis [3]. Small drug molecules can inhibit or activate caspases [4]. In apoptosis, cell shrinks, deformed, and looses its contacts to neighboring cells that resulted into fragmented cells called "apoptotic bodies" [5]. Human body removes apoptotic bodies without causing any inflammatory response. Process of apoptosis is controlled by a diverse range of cell signals [6]. In humans during cell development, many cells are produced by mitosis in excess which eventually undergo programmed cell death and their by contribute to sculpturing many organs and tissues [7]. Apoptosis is an essential process for the maintenance of tissue homeostasis. During the last decade, a number of compounds are identified to induce apoptosis. Compounds that promote apoptosis are considered as important medicaments for the treatment of cancer. Anticancer efficacy of many therapeutic agents is correlated to their apoptosis inducer ability, so identification of apoptosis-inducing activity plays an important role in discovery and development of potential anticancer agent [8]. Many anticancer drugs like camptothecins, such as topotecan and irinotecan, [9] and vinca alkaloids, such as vincristine and vinblastine [10], kill tumors to some extent through induction of apoptosis [11]. Recently, extensive advances are achieved in the field of apoptosis-based therapeutics. Many new drug candidates are currently being developed and most of them are in clinical state as potential apoptosis inducer agents. In an effort for search of new potent apoptosis-inducing agents, we have performed 2D- and 3D-QSAR study on 4-anilinoquinozaline derivatives for quantifying the necessary structural and physicochemical requirements of this series of compounds as potent apoptosis inducer and efficacious anticancer agents.

## 2. Materials and methods

QSAR is the study of the quantitative relationship between the experimental activity of a set of compounds and their physicochemical properties using statistical methods. The experimental information associated with biological activity, which is used as dependent variables in building a QSAR model. In this study, all computational work (2D- and 3D-QSAR) was performed using Vlife MDS QSAR plus software on a HP computer with Core2 Duo processor and a window XP operating system.

### 2.1 2D-QSAR modeling and dataset

Apoptosis-inducing activity data EC_50 _(μM) were taken from the published work [12]. The experimental EC_50 _values were evaluated by Cai Sui Xiong et al. in a caspase-based HTS assay in human breast cancer cells (T47D). The negative logarithm of the measured EC_50 _(μM) [pEC_50 _= -log (EC_50_)] was used as dependent variable for 2D- and 3D-QSAR analysis and it is listed in Table 1. Since some compounds showed insignificant activity, such compounds were excluded from the dataset. Compounds were sketched using 2D draw application and converted to 3D structures. Energy minimization and geometry optimization were conducted using Merck molecular force field as force field and charge, maximum number of cycles were 1,000, convergence criterion (RMS gradient) was 0.01 and medium's dielectric constant of 1 by batch energy minimization method. Energy-minimized geometry was used for calculation of descriptors, a total of 208 2D descriptors were calculated which encoded different aspects of molecular structure and consists of electronic, thermodynamic, spatial, and structural descriptors, e.g., retention index (chi), atomic valence connectivity index (chiV), path count, chain path count, cluster, path cluster, element count, estate number, semi-empirical, molecular weight, molecular refractivity, log*P*, and topological index. Various Baumann alignment-independent (AI) descriptors were also calculated.

**Table 1:** Structure, experimental, and predicted activity of 4-anilinoquinozaline derivatives


**S. no**.	**R**	**R_1_**	**R_2_**	**R_3_**	**R_4_**	**A**	**B**	**D**	**EC_50 _(μm)^a^**	**ExperimentalpEC_50_^b^**	**Predicted pEC_50_**					
											
											**MLR**	**PCR**	**PLS**	**SW-FB**	**GA**	**SA**

1	H	Me	Cl	H	OMe	C	C	C	0.002	2.699	2.695	1.215	2.496	2.197	2.196	1.838
2	H	Me	OMe	H	OMe	C	C	C	0.004	2.398	2.386	1.684	2.083	2.197	2.347	2.071
3	H	Me	NMe_2_	H	OMe	C	C	C	0.015	1.823	1.716	2.152	2.01	1.107	0.865	1.422
4	H	Me	NHMe	H	OMe	C	C	C	0.008	2.097	2.386	1.684	2.083	2.215	1.37	2.229
5	H	Me	NHNH_2_	H	OMe	C	C	C	0.023	1.639	1.441	1.215	1.216	1.052	1.755	2.431
6	H	Me	NMeAc	H	OMe	C	C	C	0.009	2.046	2.143	2.62	2.576	2.244	2.017	2.255
7	H	Me	Me	H	OMe	C	C	C	0.002	2.699	1.924	1.684	1.814	2.37	1.875	1.174
8	H	Me	Et	H	OMe	C	C	C	0.009	2.046	3.331	2.152	1.969	1.717	2.482	1.728
9	H	Me	CH_2_F	H	OMe	C	C	C	0.002	2.699	3.484	1.684	3.567	1.991	0.95	1.964
10	H	Me	CH_2_Cl	H	OMe	C	C	C	0.048	1.319	1.315	1.684	1.679	2.104	1.507	1.23
11	H	Me	CH_2_OH	H	OMe	C	C	C	0.002	2.699	2.386	1.684	2.307	2.398	1.85	2.699
12	H	Me	CH_2_NMe_2_	H	OMe	C	C	C	1.8	-0.255	0.158	1.684	-0.061	0.366	-0.338	-0.208
13	H	H	Me	H	OMe	C	C	C	6.4	-0.806	0.242	0.512	0.048	-0.066	0.251	-0.113
14	H	Me	Me	H	NO_2_	C	C	C	0.74	0.131	-0.074	1.215	0.377	-0.546	-0.23	0.628
15	H	Me	Me	H	F	C	C	C	0.42	0.377	0.518	1.215	0.679	0.986	1.541	1.711
16	F	Me	Me	H	OEt	C	C	C	0.004	2.398	2.869	2.152	1.872	2.071	2.351	2.071
17	H	Me	Me	H	OCHF_2_	C	C	C	0.009	2.046	1.892	1.684	1.833	1.559	0.129	1.238
18	H	Me	Me	H	SMe	C	C	C	0.004	2.398	1.924	1.684	1.814	1.583	1.851	2.226
19	H	Me	Me	H	Et	C	C	C	0.031	1.509	2.397	1.918	0.976	1.878	1.355	1.222
20	H	Me	Me	H	NMe_2_	C	C	C	0.016	1.796	1.815	2.152	1.742	1.731	1.615	0.927
21	H	Me	Me	H	OH	C	C	C	0.086	1.066	0.518	1.215	0.679	0.561	1.64	1.299
22	H	Me	Me	H	NH_2_	C	C	C	0.18	0.745	1.079	1.215	0.679	0.78	2.02	1.40
23	H	Me	Me	H	N_3_	C	C	C	0.011	1.959	2.102	1.215	2.055	1.433	1.913	2.58
24	H	Me	Me	H	NHAc	C	C	C	0.059	1.229	1.382	1.918	1.331	0.56	1.419	1.31
25	H	Me	Me	H	OMe	C	C	C	0.002	2.699	1.391	1.684	1.514	1.766	1.543	1.732
26	H	Me	Me	H	OMe	C	C	C	0.004	2.398	1.903	1.684	1.821	1.667	1.289	2.02
27	H	Me	Me	H	OMe	C	C	C	0.010	2	2.164	2.152	2.271	2.248	2.551	2.252
28	H	Me	Me	H	OMe	N	C	C	0.011	1.824	1.541	1.215	1.316	1.217	0.865	1.413
29	H	Me	Me	H	OMe	C	N	C	0.016	1.357	0.979	1.215	1.316	1.591	1.355	2.174
30	H	Me	Me	H	NMe_2_	N	C	C	0.015	1.482	1.432	1.684	1.244	0.989	1.293	1.18
31	H	Me	Me	H	OMe	N	C	N	0.053	1.125	0.596	0.747	0.819	1.663	2.329	1.277
32	H	Me	Me	OMe	OMe	C	N	N	0.15	0.824	0.035	0.747	0.819	1.901	1.54	1.433

^a^Data are the mean of three or more experiments and are reported as mean ± SE of the mean (SEM).

^b^The negative logarithm of the measured EC_50 _(μM).

### 2.2. Selection of training and test set

The dataset of 32 molecules was divided into training set (24 compounds) and test set (8 compounds) by Sphere Exclusion (SE) method [13] for multiple linear regression (MLR), principal component regression (PCR), and partial least squares (PLS) model with dissimilarity value of 3.2 using pEC_50 _activity field as dependent variable and various 2D descriptors as independent variables (Additional file 1). The unicolumn statistics of test and training sets (Table 2) showed the accurate selection of test and training sets, as the maximum of the training set was more than that of the test set and the minimum of the training set was less than or equal to that of the test set.

**Table 2:** Unicolumn statistics of training and test sets for apoptosis inducing activity

Set	Average	Max	Min	**Std dev**.	Sum
2D					
Training	1.595	2.699	-0.806	0.975	38.273
Test	1.774	2.699	0.824	0.632	14.189
3D					
Training	1.477	2.699	-0.806	0.918	35.449
Test	2.127	2.699	0.824	0.648	17.012

### 2.3. Regression analysis

Dataset of 32 molecules was subjected to regression analysis using MLR, PCR, and PLS as model building methods. QSAR models were generated using pEC_50 _values as the dependent variable and various descriptors values as independent variables. The cross-correlation limit was set at 0.5, number of variables in the final equation at six in MLR and five in PCR and six in PLS, and term selection criteria as *r*^2^, *F*-test 'in,' at 4 and 'out' at 3.99, *r*^2^, and *F*-test. Variance cutoff was set at 0, scaling to autoscaling, and number of random iterations to 10. Statistical measures were used for the evaluation of QSAR models were the number of compounds in regression *n*, regression coefficient *r^2^*, number of descriptors in a model *k*, *F*-test (Fisher test value) for statistical significance *F*, cross-validated correlation coefficient *q*^2^, predictive squared correlation coefficients pred_*r^2^*, coefficient of correlation of predicted data set pred_*r^2^se *and standard error (SE) of estimation *r^2 ^se *and *q*^2 ^*se*.

### 2.4. MLR analysis

MLR is a method used for modeling linear relationship between a dependent variable *Y *(pEC_50_) and independent variable *X *(2D descriptors). MLR is based on least squares: the model is fit such that sum-of-squares of differences of observed and a predicted value is minimized. MLR estimates values of regression coefficients (*r*^2^) by applying least squares curve fitting method. The model creates a relationship in the form of a straight line (linear) that best approximates all the individual data points. In regression analysis, conditional mean of dependant variable (pEC_50_) *Y *depends on (descriptors) *X*. MLR analysis extends this idea to include more than one independent variable.

Regression equation takes the form

Y=b1*x1+b2*x2+b3*x3+c

where *Y *is dependent variable, '*b*'s are regression coefficients for corresponding '*x*'s (independent variable), '*c*' is a regression constant or intercept [14,15].

### 2.5 PCR method

PCR is a data compression method based on the correlation among dependent and independent variables. PCR provides a method for finding structure in datasets. Its aim is to group correlated variables, replacing the original descriptors by new set called principal components (PCs). These PCs uncorrelated and are built as a simple linear combination of original variables. It rotates the data into a new set of axes such that first few axes reflect most of the variations within the data. First PC (PC_1_) is defined in the direction of maximum variance of the whole dataset. Second PC (PC_2_) is the direction that describes the maximum variance in orthogonal subspace to PC_1_. Subsequent components are taken orthogonal to those previously chosen and describe maximum of remaining variance, by plotting the data on new set of axes, it can spot major underlying structures automatically. Value of each point, when rotated to a given axis, is called the PC value. PCA selects a new set of axes for the data. These are selected in decreasing order of variance within the data. Purpose of principal component PCR is the estimation of values of a dependent variable on the basis of selected PCs of independent variables [16].

### 2.6 PLS regression method

PLS analysis is a popular regression technique which can be used to relate one or more dependent variable (*Y*) to several independent (*X*) variables. PLS relates a matrix *Y *of dependent variables to a matrix *X *of molecular structure descriptors. PLS is useful in situations where the number of independent variables exceeds the number of observation, when *X *data contain colinearties or when *N *is less than 5 *M*, where *N *is number of compound and *M *is number of dependant variable. PLS creates orthogonal components using existing correlations between independent variables and corresponding outputs while also keeping most of the variance of independent variables. Main aim of PLS regression is to predict the activity (*Y*) from *X *and to describe their common structure [17]. PLS is probably the least restrictive of various multivariate extensions of MLR model. PLS is a method for constructing predictive models when factors are many and highly collinear.

### 2.7. Validation of QSAR model

**Figure 1:**
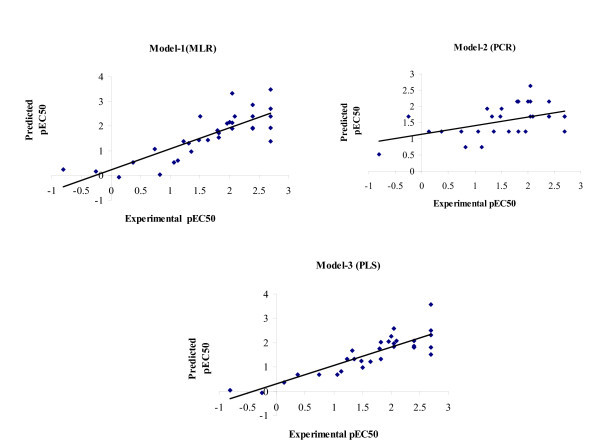
**Graphs of experimental versus predicted pEC_50 _using model-1, 2, and 3 (2D-QSAR)**.

**Figure 2:**
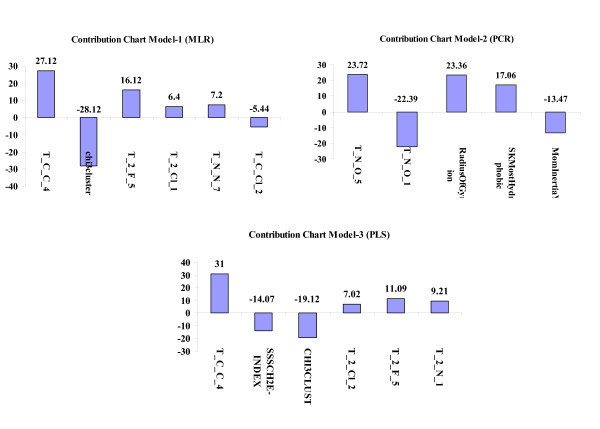
**Contribution charts of the 2D-QSAR models**.

The best way to evaluate quality of regression model is internal validation of QSAR model. Mostly leave-one-out (LOO) cross validation, one object (one biological activity value) is eliminated from training set and training dataset is divided into subsets (number of subsets = number of data points) of equal size. Model is build using these subsets and dependent variable value of the data point that was not included in the subset is determined, which is a predicted value. Mean of predicted will be same for *r*^2 ^and LOO *q*^2 ^(cross-validated correlation coefficient value) since all the data point will be sequentially considered as predicted in LOO subset. Same procedure is repeated after elimination of another object until all objects have been eliminated once. LOO cross validation resulted in three statistically significant models for each regression method [18]. To calculate *q*^2 ^following equation was used.

$$q^{2}=1-\Sigma {\left(Y_{\text{pred}}-Y_{\text{act}}\right)}^{2}∕\Sigma \left(Y_{\text{act}}-Y_{\text{mean}}\right)$$

where *Y*_pred_, *Y*_act_, and *Y*_mean _are predicted, actual, and mean values of the pIC_50_, respectively. Σ(*Y*_pred _- *Y*_act_)^2 ^is the predictive residual error sum of squares (PRESS). Definitive validity of model is examined by mean of external validation also, which evaluates how well equation generalizes. Training set is used to derive an adjustment model that is used after to predict activities of test set members. The predicted power of equations was validated using cross-validated squared correlation coefficient (*q*^2^) and by predictive squared correlation coefficients pred_*r*^2 ^which is used as a diagnostic tool. To calculate the predictive *r*^2 ^(*r*^2^_pred_) following equation was used.

$$\text{pred}\text{\_}r^{2}=1-\Sigma {\left(Y_{\text{pred}\left(\text{Test}\right)}-Y_{\text{Test}}\right)}^{2}∕\Sigma \left(Y_{\text{Test}}-Y_{\text{Training}}\right)$$

where *Y*_Pred(Test) _and *Y*_Test _are predicted and observed activity values, respectively, of test set compounds, and *Y*_Training _is the mean activity value of training set. Statistical significance of these models was further supported by 'fitness plot' obtained for each model; this is a plot of experimental versus predicted activity of training and test set compounds and provides an idea about how fit the model was trained and how well it predicts activity of external test set (Figure 1). Nearness of experimental to predicted activity reported in Table 1 also adds to this fact. Contribution charts for all the significant models are presented in Figure 2, which gives percentage contribution of descriptors used in deriving the QSAR models.

### 2.8. 3D-QSAR modeling and dataset

Dataset of 32 molecules was divided into training (24 compounds) and test (8 compounds) set by SE method having dissimilarities values of 7.9 with pEC_50 _activity field as dependent variable and various 3D descriptors calculated for the compounds as independent variables (Additional file 2).

### 2.9. Molecular modeling and alignment

Conformational search was carried out by systemic conformational search method (grid search), which generates all possible conformations, by systematically varying each of the torsion angles of a molecule by some increment, keeping the bond lengths and bond angles fixed and lowest energy conformers were selected. All the compounds were aligned by template-based method. In template-based alignment method, a template structure was defined and used as a basis for alignment of a set of molecules. In this study, all the compounds were aligned against minimum energy conformation of most active compound number using quinazoline ring as template.

### 2.10. Calculation of field descriptors

Electrostatic and steric field descriptors were calculated with cutoffs of 10.0 kcal/mol for electrostatic and 30.0 kcal/mol for steric, and charge type was selected as by Gasteiger-Marsili [19]. The dielectric constant was set to 1.0, considering distance-dependent dielectric function. Probe setting was carbon atom with charge 1.0. A total of 2,080 field descriptors (1,040 for each electrostatic and steric) were calculated for all the compounds in separate columns. 3D-QSAR analysis was performed after exclusion of all the invariable columns, as they do not contribute to QSAR.

### 2.11. k-Nearest neighbor molecular field analysis (kNN-MFA)

The kNN methodology relies on a simple distance learning approach whereby an unknown member is classified according to the majority of its kNN in training set. The nearness is measured by an appropriate distance metric. In kNN-MFA method, several models were generated for selected members of training and test sets. Once training and test sets are generated, kNN methodology is applied to descriptors generated over the grid [20]. The steric and electrostatic interaction energies are computed at lattice points of the grid using a methyl probe of charge +1. These interaction energy values are considered for relationship generation and utilized as descriptors to decide nearness between molecules.

### 2.12. kNN-MFA with stepwise forward-backward (SW-FB) variable selection method

kNN-MFA models were developed using SW-FB method with cross-correlation limit set to 0.5 and term selection criterion as *q*^2^. *F*-test 'in' was set to 4.0, and *F*-test 'out' to 3.99. As some additional parameters, variance cutoff was set at 2 kcal/mol Å and scaling to autoscaling; additionally, kNN parameter setting was done within the range of 2-5 and prediction method was selected as the distance-based weighted average.

### 2.13. kNN-MFA with genetic algorithm (GA)

GA first described by Holland [21], mimics natural evolution and selection. In biological systems, genetic information that determines the individuality of an organism is stored in chromosomes. Chromosomes are replicated and passed onto the next generation with selection criteria depending on fitness.

### 2.14. kNN-MFA with simulated annealing (SA)

SA is the simulation of a physical process, 'annealing', which involves heating the system to a high temperature and then gradually cooling it down to a preset temperature (e.g., room temperature). During this process, the system samples possible configurations distributed according to the Boltzmann distribution so that at equilibrium, low energy states are the most populated.

## 3. Results and discussion

### 3.1. Generation of 2D-QSAR models

Descriptors used in generation of 2D-QSAR models are given in Table 3 with detail description. 2D-QSAR study of 4**-**anilinoquinozaline derivatives resulted in several QSAR models. Statistically significant QSAR models were selected for discussion.

**Table 3:** Molecular descriptors used in QSAR study

Descriptor	Description
AI descriptors	
T_C_C_4	T_C_C_4 is a count of number of carbon atoms (single, double or triple bonded) separated from any carbon atom (single or double bonded) by four bonds in a molecule (C_C_C_C_C_C)
T_2_F_5	T_2_F_5 is the count of number of double bounded atoms (i.e. any double bonded atom, T_2) separated from fluorine atom by five bonds in a molecule (C_C_C_C_C_C_F)
T_2_Cl_1	T_2_Cl_1 is the count of number of double bounded atoms (i.e. any double bonded atom, T_2) separated from chlorine atom by single bonds in a molecule (C_C_Cl)
T_N_N_7	T_N_N_7 is a count of number of nitrogen atoms (single, double or triple bonded) separated from any nitrogen atom (single or double bonded) by seven bonds in a molecule (N_C_C_C_C_C_C_C_N)
T_N_O_5	T_N_O_5 is a count of number of nitrogen atoms (single, double or triple bonded) separated from any oxygen atom (single or double bonded) by five bonds in a molecule (N_C_C_C_C_C_O)
T_N_O_1	T_N_O_1 is a count of number of nitrogen atoms (single, double or triple bonded) separated from any oxygen atom (single or double bonded) by single bonds in a molecule (N_C_O)
T_2_Cl_2	T_2_Cl_2 is the count of number of two bounded atoms (i.e. any double bonded atom, T_2) separated from chlorine atom by double bonds in a molecule (C_C_C_Cl)
T_2_N_1	T_2_N_1 is the count of number of double bounded atoms separated from nitrogen atom by one bonds in a molecule (C_C_N)
Cluster	
Chi3Cluster	Chi3Cluster which signifies simple 3^rd ^order cluster chi index in a compound contributed negatively to the model-1
Distance-based topological	
RadiusOfGyration	RadiusOfGyration signifies size descriptor for the distribution of atomic masses in a molecule.
MomInertiaY	This descriptor signifies moment of interia at *Y*-axes
Hydrophobicity SlogpK	
SKMostHydrophobic	SKMostHydrophobic is the most hydrophobic value on the van der Wall surface (vdWSA). VdWSA is the surface of the union of the spherical atomic surfaces defined by the van der Waal radius of each component atom in the molecule
Electrotopological state	
SssCH2E-index	SssCH2E-index indices for number of -CH_2 _group connected with two single bonds

Model-1 (MLR)

pEC_50 _= +0.4723 (T_C_C_4) - 5.4583 (chi3Cluster) + 0.5491 (T_2_F_5) + 1.7150 (T_2_Cl_1) + 0.5612(T_N_N_7) - 1.0714 (T_C_Cl_2) + 0.0043

where *n *= 24_training _and 8_test_, DF = 17, *r*^2 ^= 0.823, *q*^2 ^= 0.763, *F*-test = 23.133, *r*^2 ^se = 0.432, *q*^2 ^se = 0.417, pred_*r*^2 ^= 0.639

Model-2 (PCR)

pEC_50 _= +0.1170 5 (T_N_O_5) - 0.2894 (T_N_O_1) + 0.5052 (RadiusOfGyration) + 1.4150 (SKMostHydrophobic) - 1.0714 (MomInertiaY) - 2.3106

where *n *= 24_training _and 8_test _, DF = 19, *r*^2 ^= 0.754, *q*^2 ^= 0.667, *F*-test = 24.549, *r*^2 ^se = 0.393, *q*^2 ^se = 0.578, pred_*r*^2 ^= 0.513

Model-3 (PLS)

pEC_50 _= +0.4335 (T_C_C_4) - 1.2055 (SssCH2E-index) - 3.1751 (chi3Cluster) + 0.7742 (T_2_Cl_1) + 0.3287 (T_2_F_5) + 0.3692 (T_2_N_1) - 3.0621

where *n *= 24_training _and 8_test _, DF = 30, *r*^2 ^= 0.824, *q*^2 ^= 0.622, *F*-test = 31.137, *r*^2 ^se = 0.435, *q*^2 ^se = 0.515, pred_*r*^2 ^= 0.589

In above QSAR models, *r*^2 ^is a correlation coefficient that has been multiplied by 100 gives explained variance in biological activity. Predictive ability of generated QSAR models was evaluated by *q*^2 ^employing LOO method. *F *value reflects ratio of variance explained by models and variance due to error in regression. High *F *value indicates that model is statistically significant. Low SE of estimation indicted by *r*^2 ^se and *q*^2 ^se suggested that models are statistically significant. Predictive ability of QSAR model was also confirmed by external validation of test set compounds denoted by pred_*r*^2^, and it was found in agreement with accepted criteria of more than 0.3. Among these three models, PLS has come out with very good results as compare to other two models. Results of PLS analysis showed very good predictive ability as indicted by *r*^2^, *q*^2^, *F*-test, and pred_*r*^2 ^values (Table 4).

**Table 4:** Statistical results of 2D-QSAR models

**S. no**.	Statistical Parameters	Model-1 (MLR)	Model-2 (PCR)	Model-3 (PLS)
1	*n*	24_Training _and 8_Test_	24_Training _and 8_Test_	24_Training _and 8_Test_
2	DF	17	19	30
3	*r*^2^	0.823	0.754	0.824
4	*q*^2^	0.763	0.667	0.6224
5	*F *test	23.133	24.549	31.137
6	*r*^2 ^se	0.432	0.393	0.435
7	*q*^2 ^se	0.417	0.578	0.515
8	pred_*r*^2^	0.639	0.513	0.589
9	pred_*r*^2^se	0.561	0.562	0.648

DF, degree of freedom; MLR, multiple linear regression; *n*, number of molecules of training set; PCR, principal component regression; PLS, partial least squares; pred_*r*^2^se, coefficient of correlation of predicted data set; *r*^2^, coefficient of determination; *q*^2^, cross-validated *r*^2^; pred_*r*^2^, *r*^2 ^for external test set.

### 3.2. Interpretaion of 2D-QSAR models

MLR (Model-1) and PLS (Model-3) indicate positive contribution of Baumann's [22] AI topological descriptors T_C_C_4, T_2_F_5, T_2_Cl_1 and negative contribution of chi3Cluster where as PCR model indicates positive contribution of RadiusOfGyration along with SKMostHydrophobic and negative contribution of T_N_O_1 and MomInertiaY.

AI descriptors can be generated considering topology of the molecule, atom type, and bond. For calculation of AI descriptors, every atom in the molecule was assigned at least one and at most three attributes. First attribute is 'T-attribute' to thoroughly characterize topology of the molecule. Second attribute is atom type, atom symbol is used here. Third attribute is assigned to atoms taking part in a double or triple bond. After all atoms have been assigned their respective attributes, selective distance count statistics for all combinations of different attributes are computed. A selective distance count statistic 'XY2' (e.g., TOPO2N3) counts all the fragments between start atom with attribute '*X*' (e.g., '2' double-bonded atom) and end atom with attribute '*Y*' (e.g., 'N') separated by graph distance 3. Graph distance can be defined as the smallest number of atoms along the path connecting two atoms in molecular structure. In this study, to calculate AI descriptors, we have used following attributes: 2 (double-bonded atom), 3 (triple-bonded atom), C, N, O, S, H, F, Cl, and Br the distance range of 0 to 7. RadiusOfGyration and MomInertiaY are distance-based topological descriptors. Topological indices are numerical values associated with chemical constitutions for the purpose of correlating chemical structure with biological activity. Distance-based topological descriptors are defined by their atom types and topological distance which signifies basic connectivity of atoms in the molecules. SKMostHydrophobic is a thermodynamic descriptor to characterize the hydrophobicity of a molecule. SlogP estimates log*P *by summing the contribution of atom-weighted solvent accessible surface areas and correction factors. SKMostHydrophobic contributed positively to the PCR model means the group, which increases hydrophobic nature, may cause increase apoptosis inducing activity. An estate contribution descriptor SssCH2E-index, which represents the electro-topological state indices for number of -CH_2 _group connected with two single bonds, is inversely proportional to the activity.

### 3.3. Generation and interpretaion of 3D-QSAR models

3D-QSAR modeling was performed using kNN-MFA method that adopts a kNN principle for generating relationships between molecular fields and apoptosis-inducing activity. The kNN-MFA models (4-6) were generated using training set of 24 compounds and 3D-QSAR models were validated using a test set of 8 compounds. The steric (S) and electrostatic (E) descriptors specify the regions, where variation in the structural features of different compounds in training set leads to increase or decrease in activities. The number accompanied by descriptors represents its position in 3D MFA grid. The stepwise forward backward variable selection method resulted in several statistically significant models, of which model-4 is considered as the best one. The model selection criterion is the value of *q*^2^, internal predictive ability of model, and that of pred_*r*^2^, ability of the model to predict activity of external test set. Model-4 (SW-FB)

pEC_50 _= E_895 (-0.0321, 0.0124) E_805 (0.0448, 0.1181)

Model-5 (SA)

pEC_50 _= E_509 (0.0355, 0.0337) E_875 (0.2828, 1.2756) S_526 (-0.6662, -0.4031) E_147 (0.1935, 0.2030)

Model-6 (GA)

pEC_50 _= S_477 (30.000, 30.000) S_664 (-0.0690, -0.0457)

Model-4 was considered as 3D-QSAR-model (SW-FB) for the dataset. Model-4 is used for internal predictivity, the value of LOO cross-validation squared correlation coefficient *q*^2 ^= 0.653 suggested goodness of the prediction. Model having predictive squared correlation coefficient (pred_*r*^2^) > 0.52, in agreement with the accepted criteria as more than 0.4 which means 52% predictive power for the external test set (Table 5).

**Table 5:** Statistical results of kNN-MFA method

**S. no**.	Statistical parameters	Model-4 (SW-FB)	Model-5 (SA)	Model-6 (GA)
1	*n*	24_Training _and 8_Test_	24_Training _and 8_Test_	24_Training _and 8_Test_
2	*k*	2	2	2
3	DF	21	19	19
4	*q*^2^	0.653	0.693	0.622
5	*q*^2^_se	0.54	0.526	0.516
6	pred_*r*^2^	0.523	0.537	0.563
7	pred_*r*^2^se	0.423	0.83	0.488
8	Descriptors (Vn)	E_895	E_509	S_477
		E_805	E_875	S_664
			S_526	
			E_147	

*n*, number of observations (molecules); k, number of nearest neighbors; DF, degree of freedom; Vn, *q*^2^, cross-validated *r*^2 ^(by the LOO method); pred_*r*^2^, predicted *r*^2 ^for the external test set; pred_*r*^2 ^se = coefficient of correlation of predicted data set; *V_n_*, number of descriptors.

**Figure 3:**
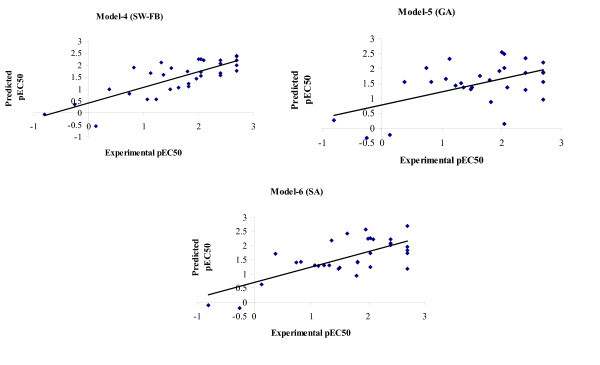
**Graphs of experimental versus predicted pEC_50 _using model-4, 5, and 6 (3D-QSAR)**.

**Figure 4:**
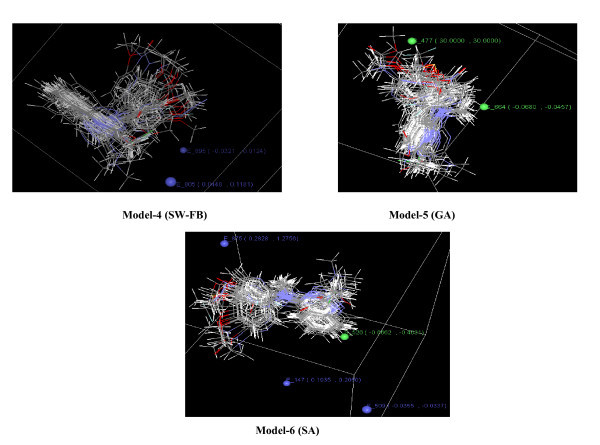
**Contour plots of 3D-QSAR models (4-6) with important steric and electrostatic points contributing to the models with range of values shown in parenthesis**.

The predicted versus the experimental value for training and test sets are depicted in Figure 3. The best 3D-QSAR (kNN-MFA) models for the prediction of apoptosis-inducing activity were obtained by applying SA giving *q*^2 ^= 0.693 and pred_*r*^2 ^= 0.537, and GA giving *q*^2 ^= 0.622 and pred_*r*^2 ^= 0.563. 3D-QSAR models (4-6) obtained showed that electrostatic and steric interactions plays major role in determination of apoptosis inducing activity. E_895 and E_805 in model-4, E_509, E_875, and E_147 in model-5 are electrostatic field descriptors, similarly S_526 in model-5 and S_477 and S_664 in model-6 are steric descriptors (Figure 4). Negative value in electrostatic field descriptors indicates that negative electronic potential is required to increase apoptosis-inducing activity and more electronegative groups are preferred in that position, positive range indicates that group that imparting positive electrostatic potential is favorable for apoptosis-inducing activity so less electronegative group is preferred in that region. Similarly, negative range in steric descriptors indicates that negative steric potential is favorable for activity, and less bulky substituents group is preferred in that region, positive value of steric descriptors reveals that positive steric potential is favorable for increase in apoptosis-inducing activity of 4-anilinoquinozalines and more bulky group is preferred in that region.

## 4. Conclusions

Apoptosis plays a pivotal role in the cytotoxic activity of most chemotherapeutic drugs. QSAR modeling resulted in identification of common structural features responsible for prediction of apoptosis-inducing activity for 4-anilinoquinozaline derivatives. 2D-QSAR studies revealed that AI descriptors were major contributing descriptors. Descriptor values obtained in this study helped in quantification of the structural features of 4-anilinoquinozaline derivatives. The overall degree of prediction was found to be around 82% in case of MLR and PLS. Among the three 2D-QSAR models (MLR, PCR, and PLS), results of PLS analysis showed significant predictive power and reliability as compare to other two methods. The master grid obtained for the various kNN-MFA models showed positive value in electrostatic field descriptors, which indicates that positive electronic potential is required to increase apoptosis-inducing activity. Negative range in steric descriptors indicates that less bulky group is preferred in that region. 3D-QSAR results suggested the importance of some molecular characteristics, which should significantly affect the binding affinities of compounds. These results provide useful clues for designing novel apoptosis inducer for the treatment of cancer.

## Competing interests

The authors declare that they have no competing interests.

## Supplementary Material

Additional file 1 **Table listing values of 2D descriptors of 4-anilinoquinozaline derivatives used in QSAR modeling**. Values of 2D descriptors used in the generation of 2D QSAR models. Click here for file

Additional file 2 **Table listing 3D descriptors of 4-anilinoquinozalines derivative required for the binding affinity**. Steric and electrostatic field values required for the binding of the molecules. Click here for file

## References

[B1] Varmus H The new era in cancer research Science 2006 312 1162 1165 10.1126/science.1126758 16728627

[B2] Kingston DG Newman DJ Taxoids: cancer-fighting compounds from nature Curr Opin Drug Disc Dev 2007 10 130 144 17436548

[B3] Wyllie AH Kerr JF Currie AR Cell death: the significance of apoptosis Int Rev Cyt 1980 68 251 306 10.1016/s0074-7696(08)62312-8 7014501

[B4] Fischer U Schulze-Osthoff K New approaches and therapeutics targeting apoptosis in disease Pharmacol Rev 2005 57 187 215 10.1124/pr.57.2.6 15914467

[B5] Fuentes-Prior P Salvesen GS The protein structures that shape caspase-activity, specificity, activation and inhibition Biochem J 2004 384 201 232 10.1042/BJ20041142 15450003 PMC1134104

[B6] Green DR Kroemer G The pathophysiology of mitochondrial cell death Science 2004 305 626 629 10.1126/science.1099320 15286356

[B7] Schulze-Osthoff K Ferrari D Los M Wesselborg S Peter ME Apoptosis signaling by death receptors Eur J Biochem 1998 254 439 459 10.1046/j.1432-1327.1998.2540439.x 9688254

[B8] Fesik SW Promoting apoptosis as a strategy for cancer drug discovery Nat Rev Cancer 2000 5 876 885 10.1038/nrc1736 16239906

[B9] Grivicich I Regner A da Rocha AB Grass LB Alves PA Kayser GB Schwartsmann G Henriques JA Irinotecan/5-fluorouracil combination induces alterations in mitochondrial membrane potential and caspases on colon cancer cell lines Oncol Res 2005 15 385 392 16491956 10.3727/096504005776449680

[B10] Kolomeichuk SN Bene A Upreti M Dennis RA Lyle CS Rajasekaran M Chambers TC Induction of apoptosis by vinblastine via c-Jun autoamplification and p53-independent down-regulation of p21WAF1/CIP1 Mol Pharmacol 2008 73 128 136 10.1124/mol.108.039750 18094076

[B11] Kolenko VM Uzzo RG Bukowski R Finke JH Caspase-dependent and -independent death pathways in cancer therapy Apoptosis 2000 5 17 20 10.1023/A:1009677307458 11227486

[B12] Cai SX Sirisoma N Pervin A Zhang H Jiang S Willardsen AJ Anderson MB Mather G Pleiman CM Kasibhatla S Tseng B Drewe J Discovery of *N*-(4-Methoxyphenyl)-*N*,2-dimethylquinazolin-4-amine, a potent apoptosis inducer and efficacious anticancer agent with high blood brain barrier penetration J Med Chem 2009 52 2341 2351 10.1021/jm801315b 19296653

[B13] Hudson BD Hyde RM Ra hr E Wood J Parameter based methods for compounds selection from chemical databases Quant Struct Act Relat 1996 15 285 289 10.1002/qsar.19960150402

[B14] Croux C Joossens K Influence of observations on the misclassification probability in quadratic discriminant analysis J Multivar Anal 2005 96 348 403

[B15] Devillers J Neuronal network in QSAR and drug design 1996 Academic Press, London

[B16] Doucet JP Barbault F Xia H Panaye A Fan B Nonlinear SVM approaches to QSPR/QSAR studies and drug design Curr Comput Aided Drug Des 2007 3 263 289 10.2174/157340907782799372

[B17] Huberty CJ Applied discriminant analysis 1994 Willey, New York

[B18] Kubyani H Variable selection in QSAR studies: an evolutionary algorithm Quant Struct Act Relat 1994 13 285 294

[B19] Gasteiger J Marsili M Iterative partial equalization of orbital electronegativity--a rapid access to atomic charges Tetrahedron 1980 36 3219 3228 10.1016/0040-4020(80)80168-2

[B20] Shen M LeTiran A Xiao Y Golbraikh A Kohn H Tropsha A Quantitative structure-activity relationship analysis of functionalized amino acid anticonvulsant agents using k nearest neighbor and simulated annealing PLS methods J Med Chem 2002 4 2811 2823 10.1021/jm010488u 12061883

[B21] Holland J Adaptation in natural and artificial systems 1976 University of Michigan Press

[B22] Balaban AT Highly discriminating distance-based topological index Chem Phys Lett 1982 89 399 404 10.1016/0009-2614(82)80009-2

