# Supplementary material for: 2D and 3D-QSAR study on 4-anilinoquinozaline derivatives as potent apoptosis inducer and efficacious anticancer agent
Source: Org Med Chem Lett. 2011 Oct 4;1:13. doi: 10.1186/2191-2858-1-13 (PMC3339342; doi:10.1186/2191-2858-1-13)
Supplement: Additional file 1 — Table listing values of 2D descriptors of 4-anilinoquinozaline derivatives used in QSAR modeling. Values of 2D descriptors used in the generation of 2D QSAR models. [file 2191-2858-1-13-S1.DOC]

**Additional file 1:** Table listing values of 2D descriptors of 4-anilinoquinozalines derivatives used in QSAR modeling

| S. no. | T_2_F_5 | T_2_Cl_1 | T_C_Cl_2 | T_N_N_7 | chi3  cluster | T_N_O_5 | T_C_C_4 | SssCH2  E-index | T_2_N_1 | Radius  Of  Gyration | Mom Inertia  Y | SK Most Hydro  phobic |
| --- | --- | --- | --- | --- | --- | --- | --- | --- | --- | --- | --- | --- |
| 1 | 0.000 | 1.000 | 0.000 | 0.000 | 1.21 | 1.000 | 17.0000 | 0.000 | 2.000 | 13.8051 | 1588.52 | 0.9345 |
| 2 | 0.000 | 0.000 | 0.000 | 0.000 | 1.206 | 1.000 | 19.0000 | 0.000 | 2.000 | 12.7684 | 1335.68 | 0.916 |
| 3 | 0.000 | 0.000 | 0.000 | 0.000 | 1.502 | 1.000 | 21.0000 | 0.000 | 2.000 | 13.8422 | 1762.67 | 0.838 |
| 4 | 0.000 | 0.000 | 0.000 | 0.000 | 1.206 | 1.000 | 19.0000 | 0.000 | 2.000 | 13.8744 | 1533.77 | 0.849 |
| 5 | 0.000 | 0.000 | 0.000 | 0.000 | 1.206 | 1.000 | 17.0000 | 0.000 | 2.000 | 13.8414 | 1446.95 | 0.741 |
| 6 | 0.000 | 0.000 | 0.000 | 0.000 | 1.597 | 1.000 | 23.0000 | 0.000 | 2.000 | 14.7550 | 2312.76 | 0.859 |
| 7 | 0.000 | 0.000 | 0.000 | 0.000 | 1.291 | 1.000 | 19.0000 | 0.000 | 2.000 | 12.5530 | 1097.47 | 0.921 |
| 8 | 0.000 | 0.000 | 0.000 | 0.000 | 1.206 | 1.000 | 21.0000 | 0.814 | 2.000 | 12.7758 | 1328.05 | 0.985 |
| 9 | 2.000 | 0.000 | 0.000 | 0.000 | 1.206 | 1.000 | 19.0000 | -0.686 | 2.000 | 12.9299 | 1515.69 | 0.920 |
| 10 | 0.000 | 0.000 | 1.000 | 0.000 | 1.206 | 1.000 | 19.0000 | 0.977 | 2.000 | 13.3259 | 1945.62 | 0.952 |
| 11 | 0.000 | 0.000 | 0.000 | 0.000 | 1.206 | 2.000 | 19.0000 | -0.186 | 2.000 | 13.1246 | 1408.27 | 0.788 |
| 12 | 0.000 | 0.000 | 0.000 | 0.000 | 1.614 | 1.000 | 19.0000 | 0.703 | 2.000 | 15.0133 | 3698.60 | 0.814 |
| 13 | 0.000 | 0.000 | 0.000 | 0.000 | 1.166 | 1.000 | 14.0000 | 0.000 | 2.000 | 14.1206 | 954.44 | 0.876 |
| 14 | 0.000 | 0.000 | 0.000 | 1.000 | 1.587 | 0.000 | 17.0000 | 0.000 | 3.000 | 14.4212 | 1715.04 | 0.854 |
| 15 | 0.000 | 0.000 | 0.000 | 0.000 | 1.375 | 0.000 | 17.0000 | 0.000 | 2.000 | 11.8898 | 2650.90 | 0.867 |
| 16 | 0.000 | 0.000 | 0.000 | 0.000 | 1.291 | 1.000 | 21.0000 | 0.672 | 2.000 | 15.4277 | 1334.69 | 0.931 |
| 17 | 4.000 | 0.000 | 0.000 | 0.000 | 1.699 | 1.000 | 19.0000 | 0.000 | 2.000 | 15.9225 | 1214.84 | 1.023 |
| 18 | 0.000 | 0.000 | 0.000 | 0.000 | 1.291 | 0.000 | 19.0000 | 0.000 | 2.000 | 14.6902 | 1272.72 | 1.014 |
| 19 | 0.000 | 0.000 | 0.000 | 0.000 | 1.291 | 0.000 | 20.0000 | 1.055 | 2.000 | 12.4138 | 1069.07 | 1.007 |
| 20 | 0.000 | 0.000 | 0.000 | 1.000 | 1.587 | 0.000 | 21.0000 | 0.000 | 2.000 | 13.6701 | 1163.33 | 0.948 |
| 21 | 0.000 | 0.000 | 0.000 | 0.000 | 1.375 | 1.000 | 17.0000 | 0.000 | 2.000 | 11.2699 | 1073.57 | 0.882 |
| 22 | 0.000 | 0.000 | 0.000 | 1.000 | 1.375 | 0.000 | 17.0000 | 0.000 | 2.000 | 12.0188 | 1067.98 | 0.804 |
| 23 | 0.000 | 0.000 | 0.000 | 2.000 | 1.291 | 0.000 | 17.0000 | 0.000 | 5.000 | 14.6237 | 3831.39 | 1.068 |
| 24 | 0.000 | 0.000 | 0.000 | 1.000 | 1.579 | 0.000 | 20.0000 | 0.000 | 2.000 | 16.1792 | 4966.38 | 0.986 |
| 25 | 1.000 | 0.000 | 0.000 | 0.000 | 1.489 | 1.000 | 19.0000 | 0.000 | 2.000 | 13.3250 | 3487.39 | 0.884 |
| 26 | 2.000 | 0.000 | 0.000 | 0.000 | 1.496 | 1.000 | 19.0000 | 0.000 | 2.000 | 13.0730 | 1234.16 | 0.922 |
| 27 | 0.000 | 0.000 | 0.000 | 0.000 | 1.420 | 1.000 | 21.0000 | 0.000 | 2.000 | 14.2178 | 1405.28 | 0.903 |
| 28 | 0.000 | 0.000 | 0.000 | 1.000 | 1.291 | 1.000 | 17.0000 | 0.000 | 3.000 | 12.9842 | 1303.76 | 0.767 |
| 29 | 0.000 | 0.000 | 0.000 | 0.000 | 1.291 | 1.000 | 17.0000 | 0.000 | 3.000 | 11.6379 | 1092.83 | 0.782 |
| 30 | 0.000 | 0.000 | 0.000 | 2.000 | 1.587 | 0.000 | 19.0000 | 0.000 | 3.000 | 12.8198 | 3207.71 | 0.829 |
| 31 | 0.000 | 0.000 | 0.000 | 1.000 | 1.291 | 1.000 | 15.0000 | 0.000 | 4.000 | 12.8422 | 3199.69 | 0.682 |
| 32 | 0.000 | 0.000 | 0.000 | 0.000 | 1.291 | 1.000 | 15.0000 | 0.000 | 4.000 | 12.7097 | 1275.73 | 0.661 |
