# Supplementary material for: 2D and 3D-QSAR study on 4-anilinoquinozaline derivatives as potent apoptosis inducer and efficacious anticancer agent
Source: Org Med Chem Lett. 2011 Oct 4;1:13. doi: 10.1186/2191-2858-1-13 (PMC3339342; doi:10.1186/2191-2858-1-13)
Supplement: Additional file 2 — Table listing 3D descriptors of 4-anilinoquinozalines derivative required for the binding affinity. Steric and electrostatic field values required for the binding of the molecules. [file 2191-2858-1-13-S2.DOC]

**Additional file 2:**3D descriptors of 4-anilinoquinozalines derivatives required for the binding affinity

| S. no. | E_895 | E_805 | E_509 | E_875 | E_147 | S_526 | S_664 | S_477 |
| --- | --- | --- | --- | --- | --- | --- | --- | --- |
| 1 | -0.51797 | -0.25754 | 0.00334 | -1.23516 | 0.37623 | -0.18703 | 30.00000 | -0.56191 |
| 2 | 0.07559 | -0.42982 | -0.0741 | 0.529118 | 0.54780 | -0.13216 | 30.00000 | 5.514934 |
| 3 | -0.57562 | 0.194865 | -0.0243 | -1.94447 | 0.27815 | 0.827422 | 30.00000 | -0.64496 |
| 4 | -0.47325 | 0.044752 | 0.00155 | -1.24865 | 0.40955 | -0.68610 | 24.33305 | -0.58034 |
| 5 | -0.59658 | 0.367427 | 0.15566 | -1.39822 | 0.35990 | -0.28194 | 22.54610 | -0.59687 |
| 6 | -0.51333 | 0.074832 | 0.00048 | -1.13573 | 0.16344 | -0.78217 | 10.85418 | -0.69188 |
| 7 | -0.09411 | 0.120725 | 0.05752 | 0.046479 | 0.33507 | -0.16849 | 30.00000 | 0.015336 |
| 8 | -0.03207 | 0.118099 | 0.04674 | 0.190337 | 0.48820 | 0.641242 | 30.00000 | 9.122273 |
| 9 | -0.11010 | 0.011224 | -0.07140 | -0.06345 | 0.33858 | -0.18990 | 30.00000 | -0.18987 |
| 10 | -0.12280 | -0.01209 | -0.06538 | -0.07640 | 0.29380 | -0.34076 | 30.00000 | -0.58608 |
| 11 | -0.01882 | -0.45862 | -0.0792 | 0.184030 | 0.44347 | 0.481260 | 30.00000 | 2.483272 |
| 12 | -0.71556 | 0.215212 | 0.06016 | -2.56504 | 0.28604 | -0.52649 | 30.00000 | -0.51005 |
| 13 | -0.77038 | 0.118803 | 0.01339 | -3.64905 | 0.08482 | -0.36528 | 30.00000 | -0.55588 |
| 14 | -0.87868 | 0.023183 | 0.01891 | -2.02464 | 0.35598 | -0.51047 | 30.00000 | -0.33114 |
| 15 | 0.028370 | 0.101602 | -0.0106 | 0.726768 | 0.48445 | 1.475476 | 30.00000 | -0.87294 |
| 16 | -0.50172 | 0.166106 | 0.03666 | -1.29121 | 0.20451 | -0.68608 | 30.00000 | -0.40756 |
| 17 | -0.02053 | 0.146959 | -0.0477 | -0.54337 | 0.15867 | 6.107171 | 30.00000 | -0.68849 |
| 18 | -0.32143 | 0.122031 | 0.01682 | -0.92174 | 0.23016 | -0.63741 | 30.00000 | -0.46425 |
| 19 | 0.154621 | 0.075480 | -0.0354 | 0.282767 | 0.19349 | -0.40314 | 30.00000 | 8.158047 |
| 20 | 0.211056 | 0.130597 | -0.0316 | 0.438563 | 0.08665 | 3.212862 | 30.00000 | 30.00000 |
| 21 | 0.034419 | 0.139465 | 0.03698 | 0.331226 | 0.35259 | 4.139638 | 30.00000 | 1.656226 |
| 22 | 0.014502 | 0.103469 | -0.0361 | -0.67168 | 0.24785 | 3.757209 | 23.04894 | -0.71474 |
| 23 | 0.108292 | 0.111347 | -0.0337 | 1.275592 | 0.20504 | -0.66616 | 30.00000 | -0.44512 |
| 24 | 0.033868 | 0.082315 | 0.01210 | 0.139090 | 0.23786 | -0.80659 | 30.00000 | 1.394376 |
| 25 | -0.06107 | 0.067507 | -0.1107 | 0.729994 | 0.33217 | -0.67967 | 30.00000 | -0.57789 |
| 26 | -0.26208 | -0.11246 | -0.1271 | -0.59819 | 0.28776 | -0.67991 | 22.55189 | -0.62683 |
| 27 | -0.35921 | -0.03282 | 0.00140 | -0.86671 | 0.42467 | -0.60970 | 30.00000 | -0.62243 |
| 28 | -0.23590 | 0.079388 | -0.0369 | -0.58387 | 0.23584 | -0.49391 | 30.00000 | -0.56674 |
| 29 | 0.074178 | 0.060700 | -0.0175 | 0.245486 | 0.24510 | 0.576033 | 30.00000 | 21.23495 |
| 30 | 0.012431 | 0.044782 | -0.1061 | 0.037752 | 0.45992 | -0.83833 | 30.00000 | 30.00000 |
| 31 | -0.26711 | 0.106139 | 0.01386 | -0.04832 | 0.31122 | -0.51959 | 30.00000 | -0.54123 |
| 32 | -0.17588 | 0.096962 | 0.00909 | -0.22640 | 0.16178 | 0.170372 | 30.00000 | -0.66882 |
